# Comparison of Physiological Responses and Training Load between Different CrossFit^®^ Workouts with Equalized Volume in Men and Women

**DOI:** 10.3390/life11060586

**Published:** 2021-06-20

**Authors:** Ronam Toledo, Marcelo R. Dias, Ramon Toledo, Renato Erotides, Daniel S. Pinto, Victor M. Reis, Jefferson S. Novaes, Jeferson M. Vianna, Katie M. Heinrich

**Affiliations:** 1 Faculty of Physical Education Sports, Federal University of Juiz de Fora, Juiz de Fora 36036-900, Brazil; ronamtoledo@gmail.com (R.T.); diasmr@gmail.com (M.R.D.); ramoncs2@hotmail.com (R.T.); jeferson.vianna@ufif.edu.br (J.M.V.); 2 Laboratory of Exercise Physiology and Morphofunctional Assessment, Granbery Methodist College, Juiz de Fora 36010-359, Brazil; 3 Faculty of Medicine, Federal University of Juiz de Fora, Juiz de Fora 36010-359, Brazil; renato.eferreira@gmail.com; 4 Minas College—FAMINAS, Muriaé 36880-000, Brazil; souza.daniel.p@gmail.com; 5 Research Center in Sports Sciences, Health Sciences and Human Development (CIDESD), University of Trás-os-Montes and Alto Douro, 5001–801 Vila Real, Portugal; victormachadoreis@gmail.com; 6 Department of Gymnastics, Federal University of Rio de Janeiro, Rio de Janeiro 21941–901, Brazil; jeffsnovaes@gmail.com; 7 Functional Intensity Training Laboratory, Department of Kinesiology, Kansas State University, Manhattan, KS 66506, USA

**Keywords:** conditioning, high-intensity functional training, methods, performance, physical fitness

## Abstract

The purpose of the present study was to compare the heart rate (HR), blood lactate and training load between different CrossFit^®^ workouts, with equalized total work volumes in men and women. The study included 23 individuals (13 men and 10 women) experienced in CrossFit^®^ training, who performed two workouts with different training types (as many reps as possible (AMRAP) and ‘for time’) but an equalized volume. Measurements of lactate, HR and rating of perceived exertion (RPE) were performed. The results show that there was no HR interaction between workout time and sex (*p* = 0.822; *η*^2^ = 0.006) and between workout type and sex (*p* = 0.064, *η*^2^ = 0.803). The HR significantly differed during each workout type (*p* < 0.001, *η*^2^ = 0.621), but not between the two workout types (*p* = 0.552, *η*^2^ = 0.017). Lactate showed no difference between the workout types (*p* = 0.474, *η*^2^ = 0.768), although the training load was higher (*p* = 0.033, *η*^2^ = 0.199) in women when they performed AMRAP. Altogether, the HR was not significantly different between training types or sex, while RPE, lactate and training load showed statistically significant differences depending on the group (women or men) or workout type (AMRAP or ‘for time’).

## 1. Introduction

CrossFit^®^ is a constantly varied and high-intensity functional training program that consists of a combination of different elements: cardiovascular, gymnastics and weight lifting [1,2]. A CrossFit^®^ training session is structured around joint mobility, warm-up, technical drills and a workout of the day (WOD). The execution of the WOD is typically performed with short or no intervals between exercises and repetitions and rounds [3]. Previous studies have identified several hormonal and immune responses [4,5], metabolic responses [1,6] and cardiovascular and perceptual responses [6,7] after CrossFit^®^ workouts.

Typically, the WODs are prescribed using training types to perform as many repetitions as possible (AMRAP) in a given period of time or as a set of tasks to be completed in the shortest possible time (‘for time’). These training types present positive stimuli for adaptations of aerobic and anaerobic capacity, especially AMRAP [8]. However, they seem to have the largest effect on increasing work capacity, even beyond changes in fitness [9].

A study by Kliszczewicz et al. [7] examined the cardiovascular and perceptual responses in the ‘Cindy’ benchmark WOD in CrossFit^®^ beginners. The authors found that the heart rate (HR) during training was >93% of the maximum HR, equivalent to vigorous exercise according to the American College of Sports Medicine [10]. Thus, several researchers have studied different ways of assessing the training load during CrossFit^®^ training [11,12]. HR is typically used as an internal load quantifier in the case of predominantly aerobic exercise. When exercise requires a considerable amount of anaerobic energy, other measures (e.g., rating of perceived exertion and blood lactate) are often added to the HR. The use of the effort perception scale (rating of perceived exertion; RPE) has been utilized by these studies to assess and quantify the intensity of training. The session RPE (s-RPE) has been considered a potential tool for these assessments [13] and has shown promise among CrossFit^®^ beginners [14]. Furthermore, since these workouts involve a considerable amount of anaerobic energy, the measurement of post-exercise blood lactate helps to estimate the amount of anerobic energy release.

Generally, studies on the physiological and perceptual responses during CrossFit^®^ benchmarks include workouts that have different training load characteristics, such as total time, intensity and volume [12,15,16,17]. Some WODs which are more often tested include ‘Nancy’ [18], ‘Fran’ [6,16], ‘Cindy’ [17,19], ‘Fight Gone Bad’ [6,16], ‘Filthy 50’, ‘Helen’ and ‘Grace’ [16]. These workouts include a combination of aerobic (i.e., ‘Nancy’, ‘Fight Gone Bad’, ‘Filthy 50’, ‘Helen’), body weight (i.e., ‘Fran’, ‘Cindy’, ‘Fight Gone Bad’, ‘Filthy 50’, Helen’) and weightlifting (i.e., ‘Nancy’, ‘Fran’, ‘Fight Gone Bad’, ‘Filthy 50’, ‘Helen,’ ‘Grace’) exercises; vary in time domains from a few minutes (i.e., ‘Fran,’ ‘Grace’) to over 20 min (i.e., ‘Filthy 50’); and are performed ‘for time’ (i.e., ‘Nancy’, ‘Fran’, ‘Filthy 50’, ‘Helen’, ‘Grace’) or for as AMRAP (i.e., ‘Cindy’, ‘Fight Gone Bad’). In addition, other researchers have developed and tested their own unique CrossFit^®^ style WODs [1,8]. To date, Bellar et al. [8] and Timón et al. [20] compared the AMRAP and ‘for time’ training types, but the exercises and the volume of repetitions and times differed between the workouts compared. 

Some studies have reported results separately for women and men involved in CrossFit^®^ training [21], but only one study [16] has examined differences between the responses of women and men. Determining differences by sex is of interest, as women and men complete the same workouts, although with sex-specific variations (e.g., weights, box heights, wall ball target heights). By design, CrossFit^®^ training sessions vary widely in volume and intensity. It is important to understand the differences between different types of workouts, namely, in terms of their bioenergetics. The two workouts herein are among the most popular and, moreover, are often used as benchmarks to assess CrossFitters’ abilities. However, for direct comparison between two workout styles (i.e., AMRAP and ‘for time’), no one has yet compared WODs with the total work volume (TWV) equalized between them, as well as including a comparison of responses between women and men. Thus, the aim of this study was to compare the HR, blood lactate and training load between different CrossFit^®^ workouts, with equal total work volumes. Our hypotheses were that (1) the ‘for time’ training would have higher lactate values but not higher values for the HR nor training load, and (2) men would have higher lactate values than women after both training sessions.

## 2. Materials and Methods

### 2.1. Participants

Twenty-three individuals participated in the study (26.5 ± 4.3 years, 70.1 ± 12.2 kg, 1.68 ± 0.11 m, 24.6 ± 2.2 kg/m^2^): 13 men and 10 women involved in CrossFit^®^ training for at least one year (24 ± 7.3 months), with a minimum weekly frequency of three days a week. All participants were enrolled in an affiliated training center of CrossFit, Inc. Participants could not have muscle and/or joint injuries or limitations that would have prevented them from performing the exercises in this study and could not have answered positively to any question from the Physical Activity Readiness Questionnaire—PAR-Q [22]. All participants signed an informed consent form prior to their participation according to CONEP resolution 466/2012 of the Ministry of Health, in line with the Declaration of Helsinki (2000). The procedures were approved by a local ethics committee (CAAE: 23511419.7.0000.5147).

### 2.2. Procedures

Participants were instructed to maintain their normal eating and social habits in the 24 h prior to the tests. Initially, an anthropometric evaluation (Welmy^®^ anthropometric scale) was performed. In the following days which were separated by at least 48 h, two training protocols developed for the study were carried out according to Table 1.

Each participant performed the workout on their own at a self-selected pace, and the movement patterns were judged according to the crossfit.com movement standard (https://www.crossfit.com/essentials/movements (accessed on 28 May 2021)). The TWV was calculated using the following formula: number of rounds x repetitions x load (expressed in kg). All individuals started with WOD 1, in which the maximum number of rounds was obtained, enabling a calculation of the TWV. To calculate the number of rounds for WOD 2, the inverse calculation of the TWV of the WOD 1 was performed. Recovery time between the two WODs was 72 h, and both workouts were completed at the same time of day.

Before each WOD, a specific warm-up was performed with the training movements: three sets of 10 deadlifts (only with the weight of the bar = 20 kg for men and 15 kg for women), 10 burpees and 10 kettlebell swings (with load of 12 kg for men and 8 kg for women).

### 2.3. Measures

#### 2.3.1. Heart Rate

Continuous monitoring of the HR was performed using a heart monitor (Polar^®^, RCX3, Kempele, Finland), and the HR was averaged at rest and every 15 s. To measure the resting HR, all participants remained lying in a supine position for 10 min in a quiet environment after arrival at the experiment site. The resting HR was the average between minutes 5 and 10. The maximum HR used was obtained after a maximum test of 2 km of rowing, which was also used to estimate the maximum oxygen consumption of the participants [23]. The maximum HR observed during the test was used once it exceeded 90% of the maximum HR predicted by the formula 208 – (0.7 × age) [24].

#### 2.3.2. Blood Lactate

After the first drop of blood was discarded through a transcutaneous puncture, capillary blood samples were collected on the medial side of the tip of the middle finger, using a disposable hypodermic lancet (Accu-Chek Safe-T-Pro Uno, Roche^®^, Hawthorne, FL, USA). Blood lactate was measured by photometric reflectance on a validated portable lactate analyzer (Accusport, Boehringer Mannheim—Roche^®^, Hawthorne, FL, USA). Before each WOD, the lactate analyzer was calibrated with a standard solution of known concentrations (4 mmol·L^−1^). Blood lactate ([La^−^]) was measured before and two minutes after each WOD.

#### 2.3.3. Training Load

The training load was estimated using the s-RPE product with the total training time [13]. To estimate the RPE, a modified version of the Borg CR-10 perceived effort scale was used 30 min after each WOD. Participants answered the following question: ‘From 0 to 10, how was your training session?’ The product of the calculation was expressed in arbitrary units (AU).

### 2.4. Statistical Analysis

The continuous quantitative variables were subjected to a Shapiro–Wilk normality test and Levene’s homogeneity test. Furthermore, the coefficient of variation was calculated to verify the distribution of data in each group. The HR and lactate were taken before (pre) and during each WOD, and the HR was stratified in quartiles of 25%, 50%, 75% and 100% and compared using a two-way ANOVA for repeated measures 4 (quartiles) vs. 2 (WOD), followed by Tukey’s post hoc test for multiple comparisons. The sphericity was confirmed through the Mauchly test and the effect size by *eta* squared. Considering the total sample size (*n* = 23), a post hoc analysis with the Gpower 3.0 software [25] indicated a 98% statistical power, requiring a minimum effect size of 0.35. The comparison of individual characteristics between women and men was performed using independent sample *t*-tests. A two-way ANOVA for independent samples (sex) and dependent measures (WOD) was performed, using Tukey’s post hoc test to identify significant differences. For this test, a statistical power of 96% was indicated, requiring a minimum effect size of 0.4. A Pearson correlation was performed to verify the relationship between the final time of the ‘for time’ and the number of repetitions of AMRAP with the responses of each dependent variable. A *t*-test was used to compare the area under the curve (AUC) between the two WODs. The level of statistical significance was *p* < 0.05. Analyses were performed using the SPSS software for Mac v.23 (SPSS Inc., Chicago, IL, USA).

## 3. Results

The assumptions of normality and homogeneity were confirmed with the Shapiro–Wilk and Levene’s tests, respectively. For individual data, the independent sample *t*-tests showed significant differences between women and men for body masses, height, BMI, weekly frequency and training experience, but not for age (Table 2).

**Figure 1:**
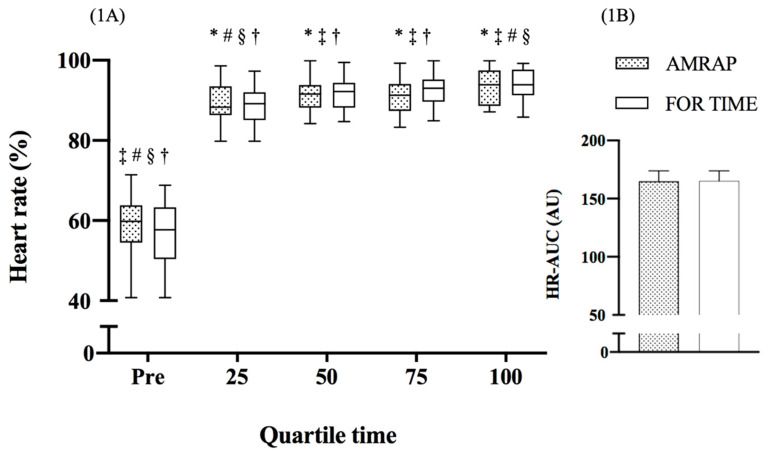
Quartiles of heart rate (HR) during the workout of the day (WOD) and area under the HR curve. Values presented in median and interquartile range (**1A**) and average and standard deviation (**1B**). HR-AUC: area under curve of heart rate. * Significant difference in relation to pre (*p* < 0.001); ^‡^ significant difference in relation to the 25th quartile (*p* < 0.01); ^#^ significant difference in relation to the 50th quartile (*p* < 0.01); ^§^ significant difference in relation to the 75th quartile (*p* < 0.01); ^†^ significant difference in relation to the 100th quartile (*p* < 0.001).

The Mauchly test found a violation of sphericity for workout time (*p* < 0.001) and the interaction of time with WOD (*p* < 0.001). There was no interaction between time and sex (F_Greenhouse-Geisser_ (1.567, 32.902) = 0.137, *p* = 0.822, *η*^2^ = 0.006) or between WOD and sex (F_Greenhouse-Geisser_ (1, 21) = 2.256, *p* = 0.064, *η*^2^ = 0.803). The HR was significantly different during the WODs (F_Greenhouse-Geisser_ (1.567, 32.902) = 34.425, *p* < 0.001, *η*^2^ = 0.621), but not between each WOD (F_Greenhouse-Geisser_ (1, 21) = 0.366, *p* = 0.552, *η*^2^ = 0.017) (Figure 1A,B, respectively). The paired *t*-test did not find differences between each WOD in HR-AUC (t(22) = −0.360; *p* = 0.722).

The two-way ANOVA showed that there were significant differences between sexes for the resting HR (F(1,21) = 5.266, *p* = 0.032, *η*^2^ = 0.200), repetitions completed (F(1,21) = 5.131, *p* = 0.034, *η*^2^ = 0.196) and TWV (F(1,21) = 44.074, *p* < 0.001, *η*^2^ = 0.677). For within-sex comparisons of responses to each WOD, significant differences were found for RPE (F(1,21) = 16.059, *p* = 0.001 *η*^2^ = 0.433), training load (F(1,21) = 9.078, *p* = 0.007, *η*^2^ = 0.302) and repetitions (F(1,21) = 14.662, *p* = 0.001, *η*^2^ = 0.411) in women, and lactate (F(1,21) = 17.402, *p* = 0.001 *η*^2^ = 0.405) and workout time (F(1,21) = 14.279, *p* = 0.001, *η*^2^ = 0.405) in men. 

Table 3 shows that greatest responses were during the ‘for time’ WOD when compared with AMRAP for RPE, training load and maximum number of repetitions (in women) and lactate (in men). Men performed the ‘for time’ WOD in less time than AMRAP. The TWV was not different between the two WODs, but men showed higher values than women for each WOD. The only statistically significant Pearson correlation coefficient was a moderate relationship for women between the maximum number of repetitions and ([La^−^]_b_) following AMRAP (*r* = 0.654; *p* = 0.040).

## 4. Discussion

The aim of this study was to compare the HR, blood lactate and training load between different CrossFit^®^ workouts with equalized total work volumes in men and women. The workouts herein are among the most popular and, moreover, are often use as benchmarks to assess CrossFitters’ ability. The main results herein partially support our a priori hypotheses. We predicted lactate values to be higher after the ‘for time’ WOD, but they were only significantly higher for men. As predicted, the HR was not different between each WOD. The training load was also hypothesized to be similar between each WOD, but for women, the training load was significantly higher in the ‘for time’ WOD. Finally, we predicted men would have higher lactate after each WOD than women, but this hypothesis was not supported.

As CrossFit^®^ training is characterized by constantly varying functional movements performed at high intensity, previous studies have investigated different types of workouts [12,18,26]. Typically, the WODs presented in the literature cover different total volumes, and that may hinder a comparison between the studies; for example: ‘Fran’ with 4 min and ‘Fight Gone Bad’ with 17 min duration [6], and ‘Cindy’ with 20 min [15,17,19] are difficult to compare with each other. The current study was the first to compare two WODs with equal volumes and to compare them between women and men. The findings of this study help us to understand the physiological and perceptual responses among WOD types by standardizing the volume of work completed by each participant.

The HR did not differ between the WODs nor between women and men. These similar responses can be explained by a similar nature of the exercises, in which the cardiac output seems to remain unchanged, likely due to the mechanical occlusion caused by muscle contractions [27]. Despite previous studies showing that the HR may not show differences between intensities during functional fitness [12], the present study attributes the absence of these differences to equalization of the TWV between the two WODs. The HR response reached 99.4% of HR_max_ during the AMRAP and 99% of HR_max_ during the ‘for time’ WODs, while the average HR over the entire workout was 91.4% and 91.7% of HR_max_ during the AMRAP and the ‘for time’ WODs, respectively. These values are in accordance with previous studies of CrossFit^®^ training [12,15,26] and with treadmill running workouts [7]. When comparing two WODs with different times (‘Cindy’ and ‘Fran’), Fernández et al. [15] did not find differences in the mean or peak HR, but they did find that ‘Cindy’ participants spent the most time in the high-intensity training zone (>80% of maximum HR). High HR values can be understood by a combination of a high demand for O_2_ as a function of muscle work and for sustaining breathing. A small difference was found in the resting HR in the WODs between women and men, which can be attributed to differences between the sexes and to intervening factors such as diet, stress and sleep quality, which quantitatively vary from 3 to 5 bpm [28,29] or up to 10 bpm [30].

When lactate was evaluated, a concentration greater than 14 mmol·L^−1^ was found after both WODs for women and men, indicating a high anaerobic output similar to that in the study by Maté-Muñoz et al. [17]. The responses in the present study showed that only men had higher blood lactate values after the ‘for time’ WOD compared with those after AMRAP. Perhaps the greater body mass and height of men and a fast start with increased exertion in the initial phase of the ‘for time’ exercise (which was observed during this study) may help to explain the higher lactate accumulation. Previous studies presented different results according to the training type. Fernández et al. [15] found no differences between the ‘Fran’ WOD (14 mmol·L^−1^) and the ‘Cindy’ WOD (14.5 mmol·L^−1^), while Tibana et al. [6] verified higher values in ‘for time’ (‘Fran’ = 17.7 mmol·L^−1^) compared with AMRAP (‘Fight Gone Bad’ = 16.2 mmol·L^−1^). Maté-Muñoz et al. [17] attributed differences in blood lactate to the recovery intervals performed during training. High lactate levels are one physiological indicator for the key aspects of the anaerobic system (e.g., the involvement of muscle fibers, the proportion of aerobic and anaerobic metabolism and the ability of glycolysis) [31]. This information is extremely relevant to determine the best training for CrossFitters and enables coaches to understand an ideal training schedule (i.e., short vs. long workouts). It is worth noting that both training types are useful, but the criteria to choose one or the other is not clear within the CrossFit^®^ methodology.

Training intensity has been assessed through various physiological and perceptual parameters [11,12,13]. Many CrossFit^®^ training sessions, both continuous and interval, are carried out with high exertion. The ability to sustain effort across the entire workout and sufficiently recover between strategized breaks would appear to be influenced by aerobic capacity [1]. In the study of Tibana et al. [12], subjects completed four rounds of a metabolic conditioning sessions designed to attain the maximum RPE (10) throughout the session. However, an RPE of 10 was attained by all participants only from round 3. In the present study, the HR increased rapidly in the first few minutes (quartile 25), remaining high during training (quartiles 50 and 75) and rising again at the end (quartile 100), which was similar for both training sessions. A progressive cardiovascular stress is typical in high-intensity exercise, either continuous or interval [20]. The results herein confirm this idea and also show that the training type (AMRAP vs. ‘for time’) did not seem to influence the HR response, since the area under the curve (AUC) did not differ between the two WOD types.

A combination of the use of external and internal loads has been suggested for the control of training performance [32], which is related to body stress and often with the need for recovery of more than 24 h [15]. However, the different training types and variation in elements in the prescription of CrossFit^®^ training make it difficult to quantify external load. Training load assessment helps coaches to choose the stress necessary for better performance and lowering injury risk. In the present study, the training load was assessed by s-RPE, 30-min after the WOD, which is a parameter found in the literature [11,12,13]. Other studies measured the RPE immediately after the session [14]. Higher RPE values were found for ‘Cindy’ than a treadmill run, but without differences in physiological stress [7]. However, when comparing ‘Cindy’ (AMRAP) with ‘Fran’ (‘for time’), no differences were found in RPE; even with different exercise times [15]. Maté-Muñoz et al. [17] compared three WODs with different elements: cardiovascular (double unders), gymnastics (‘Cindy’) and weightlifting (power cleans), and found an elevated HR and RPE during all three WODs. Lactate, measured immediately after each workout, was higher in ‘Cindy’ than in double unders but not compared to power cleans. Perhaps the combination of different movements in ‘Cindy’ (pull-ups, push-ups and squats) is a fundamental key factor for intensifying training.

In the present study, women had a higher internal load in ‘for time’ when compared with AMRAP. This difference was due to the higher s-RPE in the ‘for time’ attributed to the psychological influence of finishing the WOD in a shorter time. The TWV was balanced between the two WODs, but men showed higher values than women in both workouts, which is justified by the greater muscle mass, height, body mass index and the time of experience in CrossFit^®^. These responses agree with Billaut and Bishop [33], who demonstrated that the morphological, metabolic and neuromuscular properties produce different responses between women and men. Additionally, having a defined amount of work to complete ‘for time’ can result in participants pushing harder than when they have a set amount of time within which to complete as much work as possible [34].

It is necessary to take into account the limitations of the field testing where there is a risk of worsening or distortion of records due to the absolute load vs. the relative load. However, based on the responses to each WOD, both workout types can be recommended to develop fitness or as a part of specific sport training. In addition, our focus was on equalizing external load, and we did not account for differences in internal load. We also were unable to determine if there were ordering effects for the workouts due to the need to balance the total work volume between them. It is necessary to confirm the results from this study on a sample with different levels of conditioning and other workouts with different movement elements (cardiovascular, gymnastics and weightlifting).

## 5. Conclusions

This study demonstrated that when the workout total work volume was the same, few differences were found between the different CrossFit^®^ workout types. The heart rate did not differ between training types, nor sexes, while RPE, lactate and training load showed statistically significant differences depending on the group (women or men) or training type (AMRAP or ‘for time’). Our results indicate that coaches can use either AMRAP or ‘for time’ as strategies during CrossFit training.

## Figures and Tables

**Table 1:** CrossFit^®^ workouts tested in the study.

Characteristic	WOD ^1^ 1	WOD 2
Type	AMRAP ^2^ in 20 min	‘X’ ^3^ rounds for time
Exercise 1	13♂/11♀ calorie row on a Concept2^®^ ergometer	21♂/18♀ calorie row on a Concept2^®^ ergometer
Exercise 2	12 deadlifts at 62♂/44♀ kg	18 deadlifts at 62♂/44♀ kg
Exercise 3	10 burpees over the bar	15 burpees over the bar
Exercise 4	8 kettlebell swings at 24♂/16♀ kg	12 kettlebell swings at 24♂/16♀ kg

^1^ WOD: workout of the day. ^2^ AMRAP: as many rounds as possible. ^3^ ‘X’: number of rounds calculated to balance workout volume.

**Table 2:** Individual data between women and men who practice CrossFit^®^ training.

Characteristic	Women (*n* = 10)	Men (*n* = 13)	*p*-Value
Age (years)	25.6 ± 4.8 (18.8) ^1^	27.3 ± 3.9 (14.4)	0.359
Body mass (kg)	59.8 ± 8.6 (14.4)	78.0 ± 7.9 (10.2)	<0.001 ^2^
Height (m)	1.59 ± 0.07 (4.4)	1.75 ± 0.07 (4.0)	<0.001 ^2^
Body mass index (kg/m^2^)	23.4 ± 2.1 (8.8)	25.6 ± 1.9 (7.5)	0.016
Weekly frequency (days)	5.5 ± 0.7 (12.9)	4.6 ± 0.9 (20.8)	0.024
Training experience (months)	24.0 ± 7.4 (17.8)	31.3 ± 10.7 (34.2)	0.016

^1^ Values presented in average ± standard deviation (coefficient of variation, %). ^2^ Difference between workouts (*p* < 0.001).

**Table 3:** Comparison of dependent variables between the two workouts of the day in women and men.

Sex and Variables	As Many Rounds as Possible	For Time	*p*-Value
Women			
HR rest (bpm)	63.7 ± 7.6 (16.8) ^1^	65.8 ± 9.1 (14.7)	0.345
HR median (bpm)	170.7± 9.1 (5.3)	170.4 ± 6.7 (3.9)	0.546
HR peak (bpm)	187.7 ± 6.5 (4.7)	188.2 ± 7.7 (4.2)	0.701
Lactate (mmol·L^−1^)	16.8 ± 2.9 (32)	15.4 ± 2.6 (22.6)	0.393
RPE	7.9 ± 0.8 (16.3)	9.1 ± 0.5 (13.1)	0.001 ^2^
Time (min)	20.0 ± 0.0 (-)	19.5 ± 1.0 (5.4)	0.202
Training load (arbitrary units)	158.0 ± 17.5 (16.3)	177.9 ± 13.7 (13.9)	0.007 ^2^
Repetitions	233.0 ± 38.4 (16.5)	269.8 ± 25.6 (9.4)	0.001 ^2^
Total work volume (kg)	7281.1 ± 1363.2 (11.1)	7303.5 ± 1355 (10.1)	0.733
Men			
HR rest (bpm)	57.3 ± 9.6 (12.0) ^3^	56.6 ± 8.3 (14.0) ^3^	0.720
HR median (bpm)	177.0 ± 7.0 (4.0)	176.4 ± 8.6 (4.9)	0.647
HR peak (bpm)	183.7 ± 8.5 (3.5)	189.0 ± 7.8 (4.1)	0.637
Lactate (mmol·L^−1^)	14.1 ± 4.5 (17.3)	17.0 ± 3.8 (17.4)	0.048 ^2^
RPE	7.6 ± 1.2 (11.1)	8.1 ± 1.0 (6.2) ^3^	0.093
Time (min)	20.0 ± 0.0 (-)	18.9 ± 1.0 (1.1)	0.001 ^2^
Training load (arbitrary units)	153.8 ± 25.0 (11.1)	154.0 ± 21.4 (7.7) ^3^	0.975
Repetitions	247.3 ± 43.6 (17.6) ^3^	273.1 ± 26.8 (9.8)	0.324
Total work volume (kg)	10759.9 ± 1189.6 (18.7) ^4^	10717.3 ± 1086.8 (18.6) ^4^	0.461

^1^ Values presented in average ± standard deviation (coefficient of variation, %). ^2^ Difference between workouts (*p* < 0.05). ^3^ Difference in relation to women (*p* < 0.05). ^4^ Difference in relation to women (*p* < 0.001).

## Data Availability

Data are available on request from the study authors.
